# Survival benefit of extended lymphadenectomy in endometrial cancer: a meta-analysis with risk-stratified subgroup analysis

**DOI:** 10.3389/fonc.2026.1835770

**Published:** 2026-06-10

**Authors:** Yunlong Fan, Jun Zhu, Lingling Jiang, Jia Xu, Jinqun Huang

**Affiliations:** The First People’s Hospital of Wenling (Taizhou University Affiliated Wenling Hospital), School of Medicine, Taizhou University, Wenling, China

**Keywords:** endometrial cancer, Lymphadenectomy, overall survival, Para-aortic lymphadenectomy, risk stratification

## Abstract

**Objective:**

To systematically evaluate the effect of combined pelvic and para-aortic lymphadenectomy (PPaLND) versus pelvic lymphadenectomy alone (PLND) or no lymphadenectomy on overall survival (OS) in patients with endometrial cancer, with a focus on risk-stratified subgroup analyses to clarify whether the survival benefit varies across different recurrence risk populations, thereby providing evidence-based guidance for individualized surgical decision-making.

**Methods:**

In accordance with the PRISMA guidelines, a systematic search was conducted in PubMed, Embase, Cochrane Library, Web of Science from database inception to December 2025. Randomized controlled trials (RCTs) and cohort studies comparing no lymphadenectomy, PLND, and PPaLND that reported hazard ratios (HR) with 95% confidence intervals (CI) for OS were included. Study quality was assessed using the Newcastle-Ottawa Scale (NOS). Meta-analyses were performed using a random-effects model with Review Manager 5.4 and Stata 17.0 software. Sensitivity analysis and funnel plots were employed to assess result robustness and publication bias.

**Results:**

Fourteen studies (1 RCT, 13 retrospective studies) comprising 25,432 patients were included. Given the observational nature of the evidence, adjusted estimates were designated as primary. In unadjusted models, the effect of PPaLND on OS was inconsistent, reflecting confounding by indication. After multivariate adjustment, PPaLND was associated with a significant improvement in OS compared to no lymphadenectomy (HR = 0.61, 95% CI: 0.49–0.75). Compared to PLND, PPaLND showed a survival benefit in the overall population (HR = 0.60, 95% CI: 0.44–0.81). However, in the PPaLND versus PLND comparison, no statistically significant differences were observed in the intermediate-to-high-risk subgroup (HR = 0.62, 95% CI: 0.31–1.24), the intermediate-risk group (HR = 0.65, 95% CI: 0.01–44.47), or the high-risk group (HR = 0.43, 95% CI: 0.05–4.04); the extremely wide confidence intervals in the latter two subgroups, each based on only two studies, indicate very low statistical power, and these results should be considered exploratory. Sensitivity analysis revealed significant changes in the intermediate-to-high-risk subgroup results upon exclusion of specific studies, indicating limited robustness. Funnel plots suggested the presence of potential publication bias.

**Conclusion:**

PPaLND may confer an overall survival benefit for patients with endometrial cancer, but its value varies across different recurrence risk strata, with the survival advantage in intermediate-to-high-risk patients remaining unconfirmed. Clinical decision-making requires a careful balance between potential survival benefits and surgical complication risks. Prospective randomized controlled trials are urgently needed to validate the optimal surgical strategy.

## 1 Introduction

Endometrial carcinoma (EC) represents the most common malignancy of the female reproductive system in developed nations, with its incidence continuing to rise globally. The evaluation of lymph node involvement is a cornerstone of prognostic stratification and a critical determinant in guiding postoperative adjuvant therapy for this disease (1, 2). Surgical resection, primarily consisting of hysterectomy with bilateral salpingo-oophorectomy, constitutes the primary curative treatment modality. Over the past two decades, the paradigm of surgical management has shifted significantly toward minimally invasive approaches, including conventional laparoscopy and robot-assisted techniques. This evolution has demonstrably improved perioperative outcomes by reducing blood loss, shortening hospital stays, and accelerating recovery, without compromising oncological safety for appropriately selected patients (3).

However, within the surgical algorithm for endometrial cancer, the role and optimal extent of lymphadenectomy remain among the most contentious and debated issues. Lymph node status is integral to the FIGO surgical staging system, yet whether lymphadenectomy serves merely a diagnostic (staging) purpose or confers an independent therapeutic benefit remains unclear. The central controversy hinges on the necessity of performing a systematic para-aortic lymphadenectomy in addition to, or instead of, a standard pelvic lymphadenectomy, and the growing consideration of sentinel lymph node (SLN) mapping as a potentially less morbid alternative to comprehensive nodal dissection (4, 5). Although the FIGO committee formally incorporated surgical staging into its recommendations in 1988 (6), the therapeutic justification for lymphadenectomy—particularly the indications, anatomical boundaries, and survival impact of para-aortic nodal dissection—continues to generate significant discord within the gynecologic oncology community (7).

The body of evidence regarding the survival benefit of systematic lymphadenectomy is characterized by notable inconsistency. Seminal randomized controlled trials, such as ASTEC and others, concluded that systematic pelvic lymphadenectomy in early-stage disease did not confer a survival advantage and was associated with increased surgical morbidity (8). This challenged the therapeutic rationale for routine nodal dissection. In contrast, a substantial number of large-scale retrospective cohort studies and subsequent meta-analyses have suggested that a more extensive lymphadenectomy, encompassing the para-aortic region, may be associated with improved survival outcomes, particularly for patients deemed at intermediate or high risk of recurrence based on uterine pathological factors (9, 10). The influential SEPAL study, a retrospective cohort analysis by Todo et al., provided compelling evidence that for patients with intermediate- or high-risk endometrial cancer, combined pelvic and para-aortic lymphadenectomy (PPaLND) was associated with a significant improvement in overall survival compared to pelvic lymphadenectomy (PLND) alone (11). This finding reignited the debate and suggested a potential therapeutic role for extended dissection.

This potential benefit must be carefully weighed against the non-negligible risks associated with the procedure. Systematic lymphadenectomy, and PPaLND in particular, is linked to a distinct profile of postoperative complications. These include chronic lower-limb lymphedema, symptomatic lymphocyst formation, intraoperative vascular or neurologic injuries, and a potential increase in postoperative ileus or small bowel obstruction. Such complications can have a profound and lasting negative impact on a patient’s quality of life, and their risk may offset any marginal survival gain, especially in populations where the benefit is uncertain (12, 13).

Given this ongoing clinical equipoise and the heterogeneity of existing evidence, there is a pressing need for a contemporary, rigorous synthesis of the available data. This systematic review and meta-analysis aims to critically appraise and quantitatively synthesize the comparative evidence on the impact of different lymphadenectomy extents—specifically, no lymphadenectomy, PLND alone, and PPaLND—on overall survival in patients with endometrial carcinoma, with a particular focus on risk-stratified subgroup analyses to determine whether the survival benefit of extended lymphadenectomy varies across different recurrence risk populations. The objective is to provide an updated, high-level evidence base to inform individualized surgical decision-making and to highlight key areas for future research.

## 2 Methods

### 2.1 Study design

This investigation was designed and conducted as a systematic review and meta-analysis in strict accordance with the guidelines outlined in the Preferred Reporting Items for Systematic Reviews and Meta-Analyses (PRISMA) 2020 statement. A detailed study protocol outlining the objectives, search strategy, inclusion criteria, and planned analysis was developed *a priori* to minimize bias in the review process.

### 2.2 Data sources and search strategy

A comprehensive and systematic literature search was performed to identify all relevant studies. The following electronic bibliographic databases were queried from their inception until December 2025: PubMed, Embase, Cochrane Library, Web of Science. No initial restrictions were placed on publication language, though the search syntax was constructed in English and Chinese.

The search strategy was developed in collaboration with a medical information specialist and utilized a combination of controlled vocabulary (e.g., MeSH terms in PubMed, Emtree terms in Embase) and free-text keywords related to the population (endometrial cancer) and the intervention (lymphadenectomy). Core search concepts included: (“Endometrial Neoplasms”[Mesh] OR “endometrial cancer*” OR “cancer of the corpus uteri”) AND (“Lymph Node Excision”[Mesh] OR “Lymphadenectomy”[Mesh] OR “pelvic lymphadenectomy” OR “para-aortic lymphadenectomy” OR “lymph node dissection”). The search strategy was adapted for the syntax requirements of each database. To ensure saturation, the reference lists of all included primary studies and relevant systematic reviews were manually screened for additional eligible citations.

### 2.3 Eligibility criteria

Studies were assessed for inclusion based on the following pre-defined criteria, structured using the PICOS (Population, Intervention, Comparison, Outcome, Study design) framework:

Population: Adult women (≥18 years) with a histologically confirmed diagnosis of endometrial carcinoma of any histological subtype (endometrioid, serous, clear cell, carcinosarcoma, etc.) and any FIGO (2009 or 2023) surgical stage.
Intervention/Exposure: Surgical treatment involving one of the following lymph node management strategies: Combined pelvic and PPaLND; PLND alone; No lymphadenectomy (with or without hysterectomy).
Comparison: Studies must directly compare at least two of the three strategies listed above (e.g., PPaLND vs. PLND; PLND vs. no LND; PPaLND vs. no LND).
Outcomes: The primary outcome of interest was overall survival (OS), defined as the time from surgery or diagnosis to death from any cause. Secondary outcomes included recurrence-free survival (RFS)/disease-free survival (DFS), locoregional and distant recurrence rates, and perioperative morbidity (e.g., lymphedema, lymphocyst, vascular injury). For quantitative synthesis, studies must have reported effect estimates for survival (Hazard Ratio, HR) with measures of variance (95% Confidence Interval, CI) or provided sufficient data in text or figures (Kaplan-Meier curves) to allow for estimation of these statistics.
Study Design: Randomized controlled trials (RCTs), prospective cohort studies, or retrospective cohort studies were eligible for inclusion.
Exclusion criteria: (1) Case reports, commentaries, letters, conference abstracts, or review-type literature; (2) Studies lacking a control group or from which effect measures cannot be extracted; (3) Studies with a sample size of less than 30 cases; (4) Duplicate publications or studies with overlapping data, where only the report with the largest sample size or the most complete information was retained.

All records identified through the database searches were imported into a reference management software (EndNote X20) and duplicates were removed. The study selection process was performed independently by two reviewers (initials blinded for review). The process involved two sequential screening phases: 1) Title/Abstract Screening: Titles and abstracts were screened against the eligibility criteria. Clearly irrelevant records were excluded. 2) Full-Text Review: The full-text articles of all potentially eligible records were retrieved and assessed in detail for final inclusion.

At both stages, any disagreements between the two reviewers were resolved through discussion until consensus was reached. If consensus could not be achieved, a third senior reviewer was consulted for arbitration.

### 2.4 Data extraction and management

Data from each included study were extracted independently by the same two reviewers using a pre-piloted, standardized data extraction form created in Microsoft Excel. The extracted data included: 1) Study Identification: First author, year of publication, journal, country/countries where the study was conducted. 2) Study Characteristics: Study design (RCT, prospective/retrospective cohort), data source (single-center, multi-center, national database), study period, median follow-up time. 3) Participant Characteristics: Total sample size, number of patients in each comparison arm, mean/median age, distribution of FIGO stage, histology, tumor grade, lymphovascular space invasion (LVSI) status, and depth of myometrial invasion. 4) Intervention Details: Precise description of the surgical procedures for each arm (e.g., extent of pelvic dissection, upper limit of para-aortic dissection—inferior mesenteric artery vs. renal veins), median number of lymph nodes retrieved. 5) Outcome Data: For OS and RFS/DFS: adjusted and unadjusted Hazard Ratios (HRs) with 95% CIs. The statistical model used for adjustment and the covariates included were recorded. If HRs were not directly reported, survival probabilities at specific time points (e.g., 3-year, 5-year) and/or digitized Kaplan-Meier survival curves were extracted for subsequent estimation. Data on recurrence patterns and complication rates were extracted as reported.

### 2.5 Assessment of risk of bias

The methodological quality and risk of bias of the included studies were assessed independently by two reviewers. For randomized controlled trials, the revised Cochrane Risk of Bias tool (RoB 2.0) was planned for use. For non-randomized studies (cohort studies), the Newcastle-Ottawa Scale (NOS) was employed. The NOS assesses studies across three domains: Selection (representativeness of the exposed cohort, selection of the non-exposed cohort, ascertainment of exposure, demonstration that outcome of interest was not present at start), Comparability (comparability of cohorts on the basis of the design or analysis), and Outcome (assessment of outcome, adequacy of follow-up). A star system is used, with a maximum score of 9. Studies scoring ≥7 were considered to have a low risk of bias, scores of 5–6 indicated moderate risk, and scores ≤4 indicated high risk of bias.

Disagreements in quality assessment were resolved by consensus or third-reviewer consultation.

### 2.6 Data synthesis and statistical analysis

The primary measure of effect was the Hazard Ratio (HR) for overall survival. Where possible, adjusted HRs from multivariate Cox proportional hazards models were used for the primary meta-analysis, as they account for potential confounding factors. If a study reported only unadjusted HRs or survival rates, these data were used in secondary or sensitivity analyses.

All meta-analyses were performed using Stata software (version 17.0, StataCorp). Given the anticipated clinical and methodological heterogeneity across included studies (e.g., variations in patient risk profiles, surgical techniques, adjuvant therapy protocols, follow-up duration), a random-effects model using the DerSimonian and Laird method was chosen *a priori* for all pooled analyses. This model incorporates both within-study and between-study variance and provides a more conservative estimate when heterogeneity is present. Pooled HRs with their 95% CIs were calculated. Heterogeneity among studies was quantified using the I² statistic, with values of 25%, 50%, and 75% typically interpreted as indicating low, moderate, and high heterogeneity, respectively. The Cochran’s Q test (with a significance level of p < 0.10) was also used to assess statistical heterogeneity.

Pre-planned subgroup analyses were conducted to explore potential sources of heterogeneity and to assess the consistency of effects across different patient risk category. Risk-stratified subgroup analyses were performed using the risk categories as originally reported by each study. As the included studies employed different classification systems (e.g., ESMO-ESGO-ESTRO, GOG, or author-defined criteria), a uniform reclassification of patients across studies was not feasible without access to individual patient data.

Sensitivity analyses were performed to assess the robustness of the pooled results. The primary method was the leave-one-out analysis, iteratively removing one study at a time to evaluate its influence on the overall summary estimate. Additional sensitivity analyses were planned based on study quality (restricting to studies with NOS ≥7).

To evaluate potential publication bias, funnel plots were generated by plotting the logHR against its standard error for the primary outcome (OS) when 10 or more studies were available for a given comparison. Visual asymmetry of the funnel plot was assessed, and Egger’s linear regression test was performed to provide a statistical test for small-study effects (publication bias), with a p-value <0.05 considered suggestive of significant bias.

All statistical tests were two-sided, and a p-value <0.05 was considered statistically significant, except for tests of heterogeneity (p<0.10).

## 3 Results

### 3.1 Literature search process

**Figure 1:**
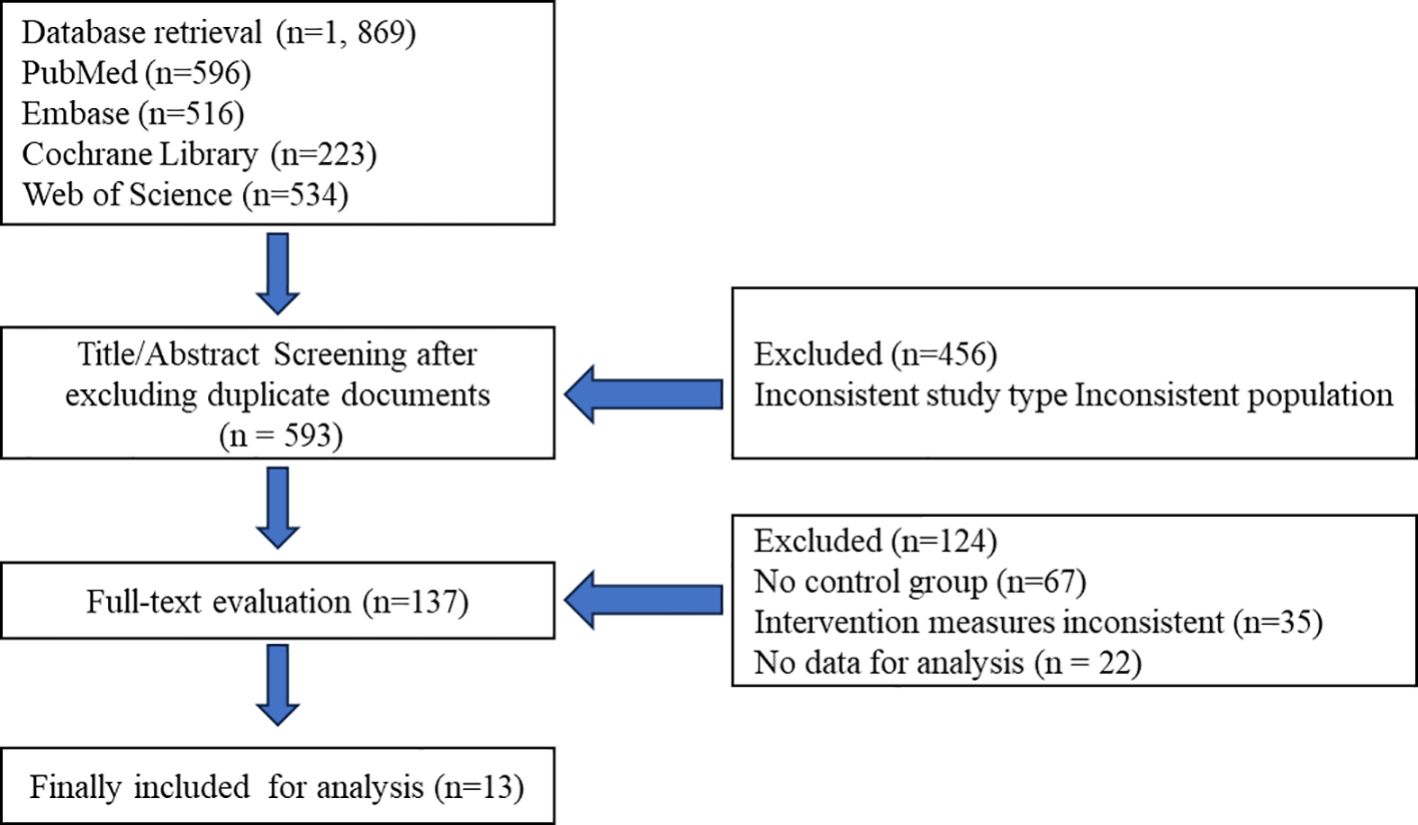
Literature screening flowchart.

The systematic search of the PubMed, Embase, Cochrane Library, and Web of Science databases initially yielded 1,869 potentially relevant citations. After removing duplicates, 593 unique records remained for title and abstract screening. Of these, 456 records were excluded primarily due to mismatched study types (e.g., reviews, case reports), irrelevant study populations, or other clear ineligibility. This resulted in 137 articles undergoing full-text review for detailed eligibility assessment. During the full-text evaluation phase, an additional 123 articles were excluded for the following reasons: lack of a comparative control group (n=67), inconsistent intervention comparisons (n=35), or insufficient data for quantitative analysis (n=21). Ultimately, 14 studies meeting all pre-defined inclusion criteria were selected for the final systematic review and meta-analysis (**Figure 1**).

### 3.2 Characteristics of included studies

This review ultimately included 14 studies published between 2008 and 2025. The body of evidence comprises one multicenter randomized controlled trial (the ASTEC trial, 2009) and 13 retrospective studies. The cumulative sample size across all included studies was 25, 432 patients, with individual study sample sizes ranging from a median of 56 to 12,333 participants. The enrolled patient populations encompassed various recurrence risk stratifications (low, intermediate, and high risk) and histological types (endometrioid and non-endometrioid carcinomas). The compared surgical interventions included combined PPaLND, PLND alone, and no lymphadenectomy. Methodological quality was assessed using the NOS, with all included studies rated as medium to high quality (scores of 6-8). (**Table 1**) Notably, among the 14 included studies, only five adjusted for both lymphovascular space invasion and adjuvant therapy in their multivariable models (**Supplementary Table 1**).

**Table 1:** Characteristics of included studies.

Author	Year	Study design	Patients grouping	Classification system/Source	Enrollments	Intervention and enrollments	Core finding	NOS
Eggemann H (14)	2016	Multicenter, retrospective study	Low, intermediate, and high risk of recurrence	Not specified (author-defined based on clinicopathological factors)	1502	No lymphadenectomy (316), Pelvic lymphadenectomy (401), PPaLND (212)	In intermediate and high risk, PPaLND significantly reduced the mortality risk in comparison with no lymphadenectomy. In low risk patients, lymphadenectomy had no effect on overall survival.	8
Toptas T (15)	2015	Singlecenter, retrospective study	Low, Low-intermediate, High-intermediate and high risk of recurrence	GOG risk of recurrence criteria	186	PLND (97), PPaLND (89)	PPaLND did not provide any survival advantage over PLND alone	8
Tong SY (16)	2011	Multicenter, retrospective study	All populations and high-risk group	Author-defined: (1) deep MI with G3, or (2) deep MI/cervical extension with any grade	547	PLND (413), PPaLND (296)	PPaLND did not provide any survival advantage over PLND alone	7
Todo Y (11)	2010	Multicenter, retrospective study	Low, intermediate, and high risk of recurrence	Not explicitly stated; appears consistent with JGOG criteria	671	PLND (325), PPaLND (346)	PPaLND is recommended as treatment for patients in intermediate or high risk of recurrence.	6
Chang SJ (17)	2008	Singlecenter, retrospective study	Low and intermediate to high risk of recurrence	Author-defined	160	PLND (75), PPaLND (85)	PPaLND is recommended as treatment for patients in intermediate to high risk of recurrence.	7
Vatansever D (18)	2020	Singlecenter, retrospective study	Early-Stage Type II Endometrial Carcinoma (Intermediate-risk)	Histology-based (inherently high-risk)	135	PLND (66), No lymphadenectomy (69)	PPaLND prolongs the survival of patients with early-stage type II endometrial carcinoma	8
Yalcin Y (19)	2025	Singlecenter, retrospective study	Early stage endometrioid grade 3 and non-endometrioid endometrial cancers (Intermediate and high-risk)	Histology- and grade-based (intermediate-to-high risk)	182	PLND (85), PPaLND (97)	PPaLND did not confer a survival advantage.	8
Seagle BL (20)	2017	Multicenter, retrospective matched study	Stage I endometrioid endometrial cancer (Intermediate risk)	Stage- and histology-based (intermediate risk)	12333	No lymphadenectomy (7487), PLND (7060), PPaLND (7060)	PPaLND prolongs the survival of patients with Stage I endometrioid endometrial cancer.	7
Lai YL (21)	2025	Multicenter, retrospective matched study	FIGO stage IIIC1 high-grade endometrial cancer (High risk)	Stage- and grade-based (high risk)	56	PLND (18), PPaLND (38)	PPaLND did not improve survival in patients with clinically FIGO IIIC1 endometrial cancer.	6
Papathemelis T (22)	2017	Single center, retrospective study	High-grade type I and II endometrial carcinoma (High risk)	Grade- and histology-based (high risk)	204	PLND (83), PPaLND (121)	PPaLND improve survival in patients with high-grade endometrial cancer.	7
Pauly L (23)	2020	Multicenter, matched cohort Study	Low, intermediate, and high risk of recurrence	ESMO guidelines	2030	PLND (1015), PPaLND (1015)	PPaLND did not reduce the risk of death compared with PLND.	8
ASTEC study group (24)	2009	Multicenter, randomized Study	Early-stage disease at intermediate or high risk	FIGO stage + grade: Low = IA/IB G1/G2; Int-High = IA/IB G3, serous, clear cell	1408	PLND (704), PPaLND (704)	No evidence of benefit in terms of overall survival for PLND in women with early endometrial cancer.	8
Venigalla S (25)	2018	Multicenter, retrospective matched study	non-endometrioid endometrial carcinoma (high risk)	Histology-based (inherently high-risk)	7250	No lymphadenectomy (930), PLND (2177), PPaLND (4143)	LND is associated with survival benefit in patients with non-endometrioid endometrial cancers.	7
Tsikouras P (26)	2016	Single center, retrospective study	intermediate to high-risk endometrioid tumors	Author-defined	270	PLND (177), PPaLND (93)	PPaLND had no better therapeutic value in women with intermediate to high risk endometrioid tumors than PLND.	6

### 3.3 Meta-analysis results

#### 3.3.1 PPaLND compared to control (No LND or PLND)

##### 3.3.1.1 Unadjusted analysis

**Figure 2:**
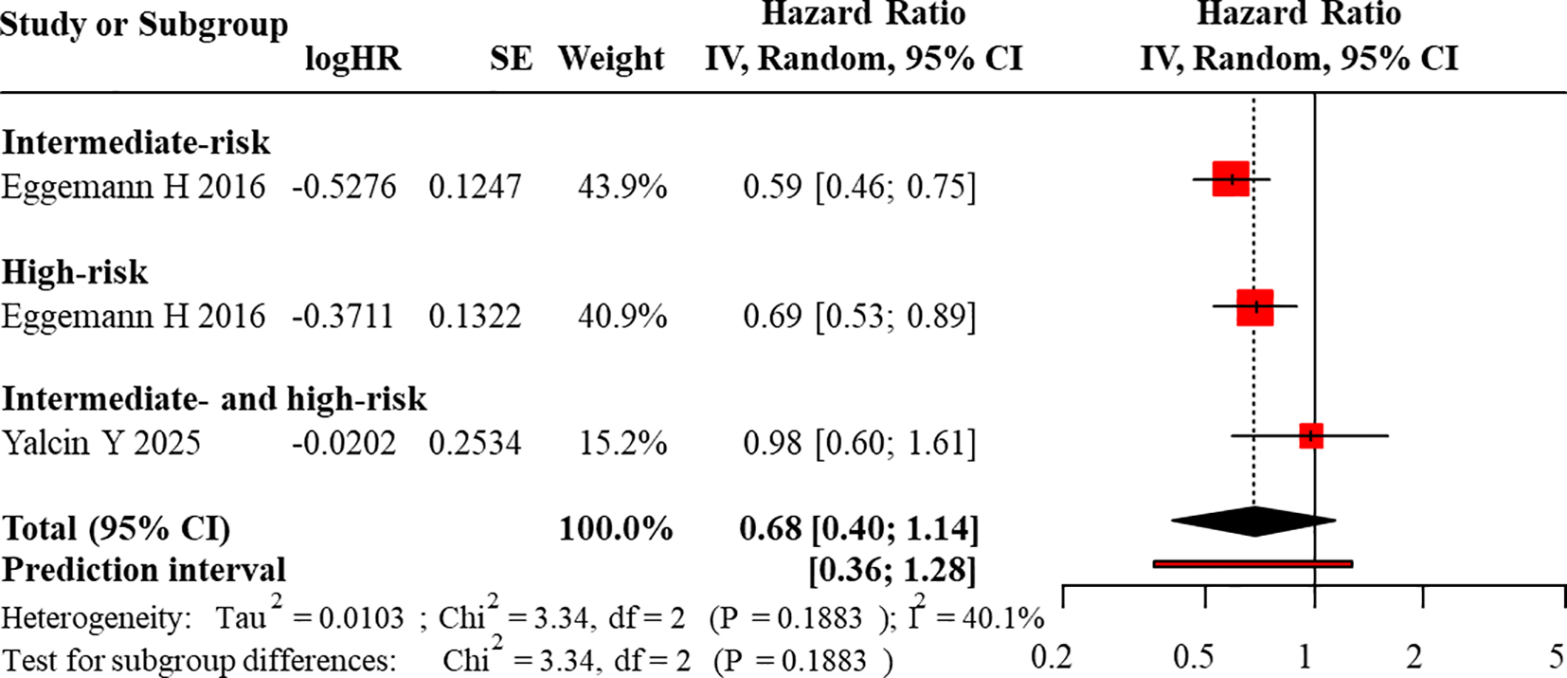
Forest plot for OS benefit of PPaLND versus control (unadjusted variables).

Two and four studies, respectively, reported on the OS benefit of PPaLND compared to control groups (comprising either no lymphadenectomy or PLND) using unadjusted models. Within these unadjusted analyses, one study reported outcomes for intermediate- and high-risk patients, while another reported for a combined intermediate-to-high-risk group. The findings were inconsistent: PPaLND was associated with increased OS benefit in intermediate- and high-risk patients but showed no such benefit in the intermediate-to-high-risk group. Pooled across all subgroups, the unadjusted analysis did not demonstrate a significant OS benefit for PPaLND (HR = 0.68, 0.40, 1.14). The forest plot for the OS benefit of PPaLND compared to control (unadjusted) is presented in **Figure 2**.

##### 3.3.1.2 Adjusted analysis

**Figure 3:**
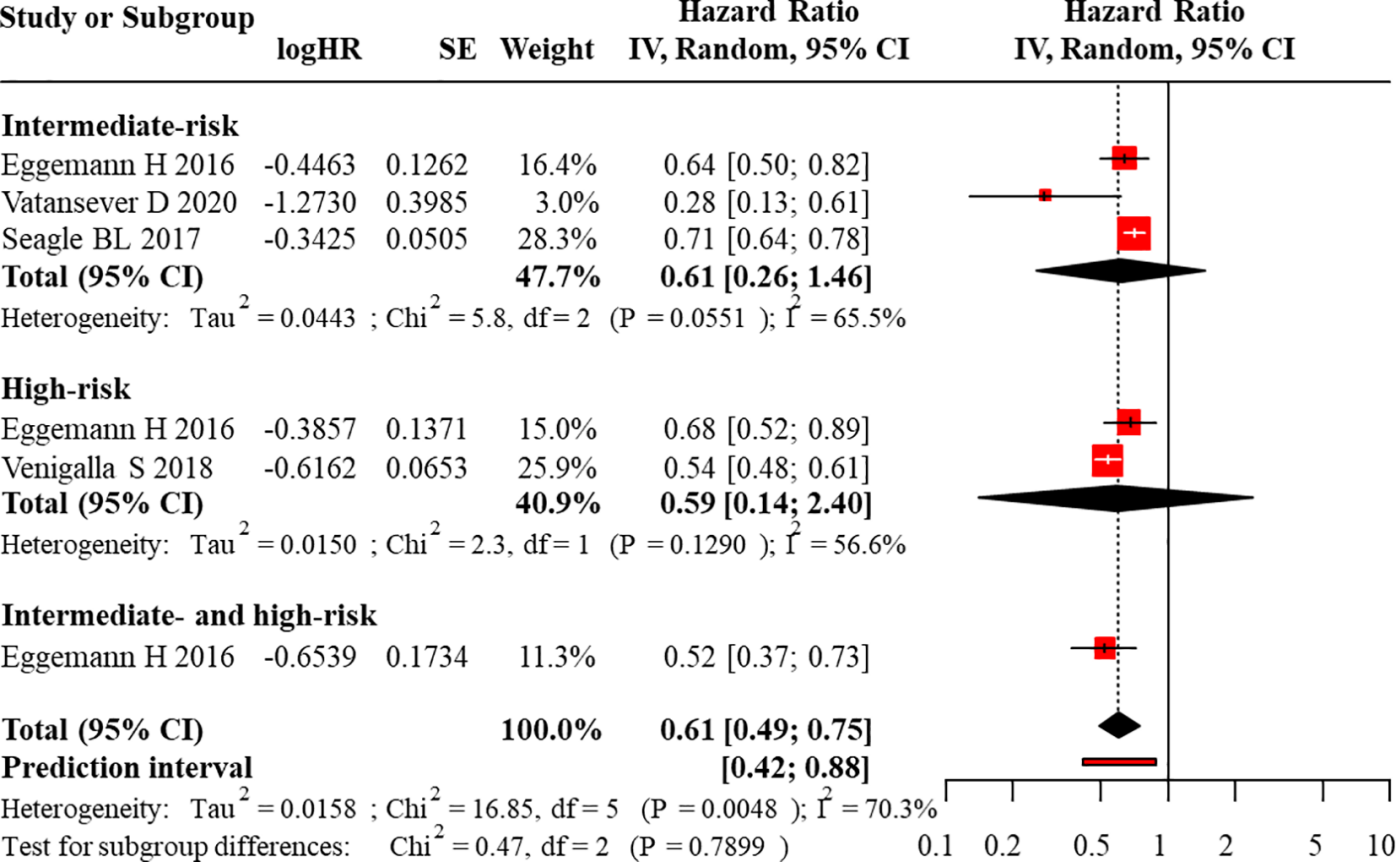
Forest plot for OS benefit of PPaLND versus control (adjusted variables).

**Figure 4:**
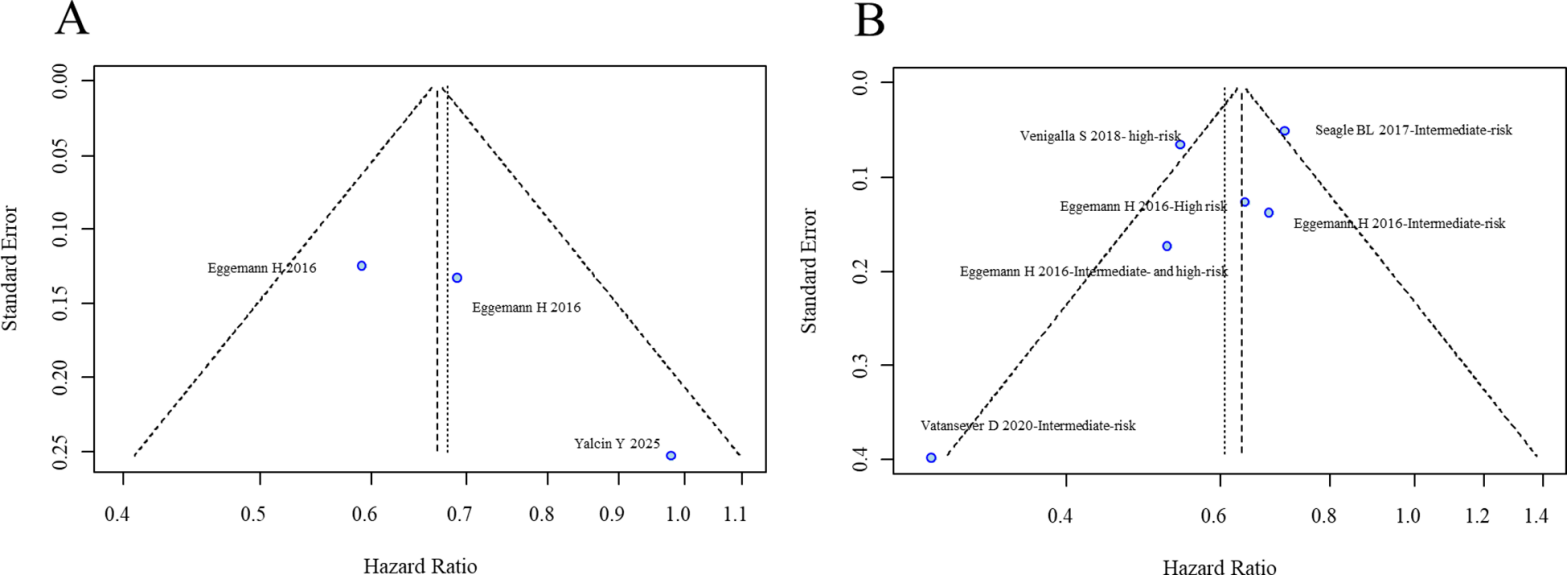
Funnel plot for OS benefit of PPaLND versus control (**A** unadjusted variables; **B** adjusted variables).

Three studies reported on the OS benefit of PPaLND compared to control using multivariable-adjusted models. Specifically, three studies reported outcomes for intermediate-risk patients, and two studies for high-risk patients. While individual studies suggested a survival benefit for PPaLND, the pooled results for these specific subgroups were not statistically significant: the HR was 0.64 (95% CI: 0.26, 1.46) for the intermediate-risk group and 0.59 (95% CI: 0.14, 2.40) for the high-risk group. For the combined intermediate-to-high-risk category, only one study provided data, reporting a significant benefit (HR = 0.52, 95% CI: 0.37, 0.73). Notably, when data from all subgroups were pooled, PPaLND was associated with a statistically significant improvement in OS (HR = 0.61, 95% CI: 0.49, 0.75). The forest plot for the adjusted analysis is shown in **Figure 3**. Assessment for publication bias using funnel plots suggested visual asymmetry and potential small-study effects (**Figure 4**). As only 3 studies contributed to this comparison, funnel plot interpretation is of limited reliability; the Cochrane Handbook recommends that tests for funnel plot asymmetry be performed only when at least 10 studies are available.

##### 3.3.1.3 Sensitivity analysis for intermediate-risk subgroup

**Figure 5:**
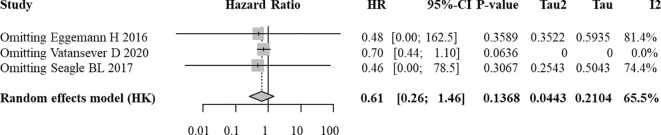
Sensitivity analysis forest plot for OS benefit of PPaLND versus control (adjusted).

For the intermediate-risk subgroup analysis (based on three studies), a leave-one-out sensitivity analysis was performed. Exclusion of the study by Vatansever D et al. (2020) (18) reduced the heterogeneity (I²) to 0% and substantially altered the pooled HR, indicating that the results for this subgroup are not robust and require validation from additional research (**Figure 5**).

#### 3.3.2 PPaLND Compared to PLND Alone

##### 3.3.2.1 Unadjusted analysis

**Figure 6:**
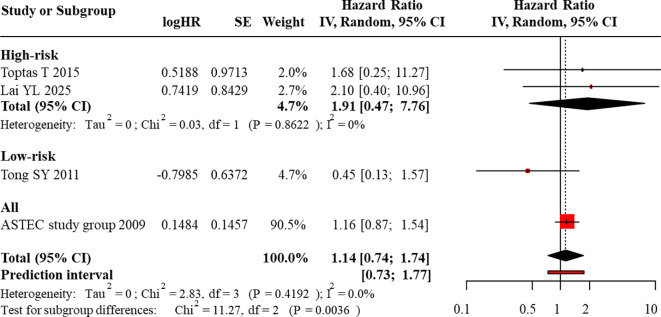
Forest plot for OS benefit of PPaLND versus pelvic (unadjusted variables).

Three and nine studies, respectively, reported unadjusted comparisons of OS between PPaLND and PLND alone. In the unadjusted model, two studies focused on high-risk patients and found no OS benefit for PPaLND over PLND. Similarly, no significant OS benefit was observed for PPaLND in low-risk patients or in the overall patient population analyzed. The forest plot for this unadjusted comparison is shown in **Figure 6**.

##### 3.3.2.2 Adjusted analysis

**Figure 7:**
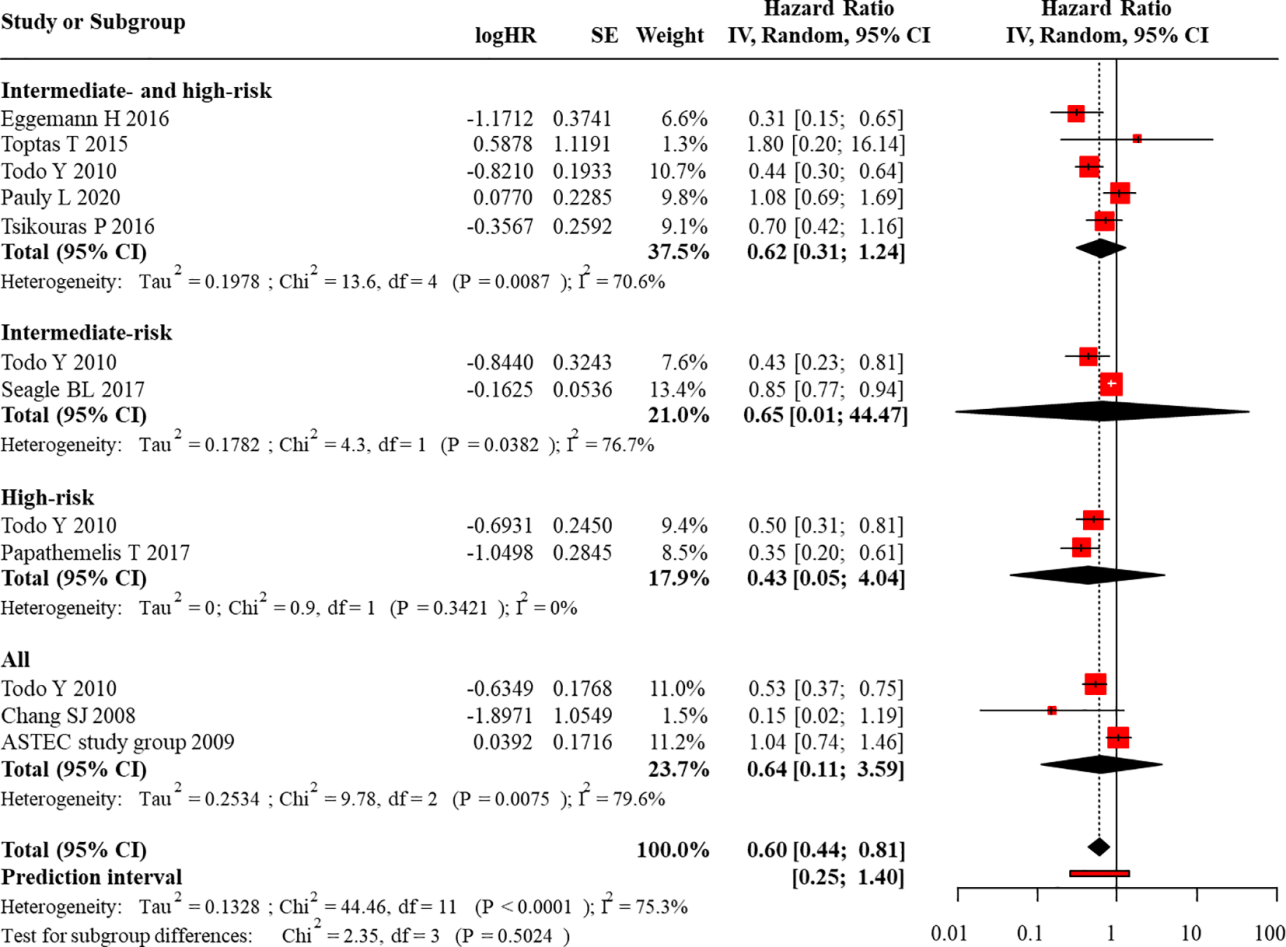
Forest plot for OS benefit of PPaLND versus pelvic (adjusted variables).

**Figure 8:**
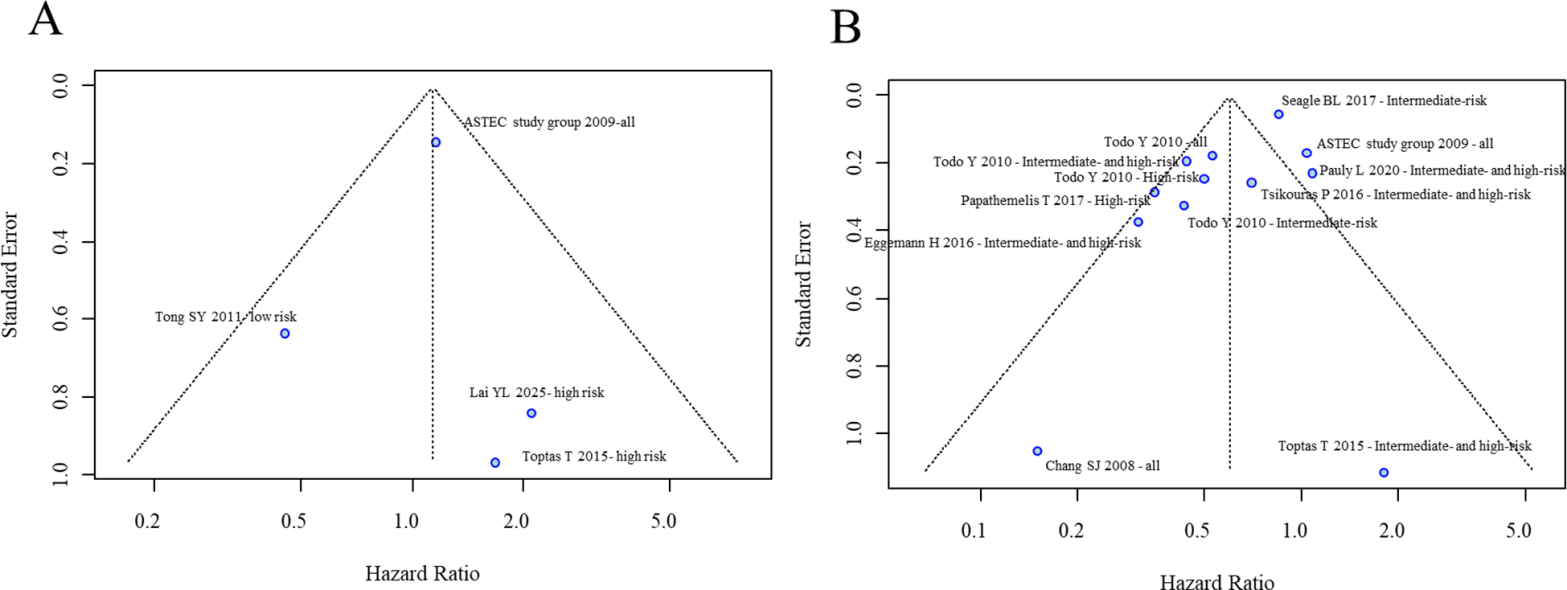
Funnel plot for OS benefit of PPaLND versus Pelvic (**A** Unadjusted variables; **B** Adjusted variables).

Nine studies provided multivariable-adjusted comparisons of OS between PPaLND and PLND. These included data for several subgroups: intermediate-to-high-risk (5 studies), intermediate-risk (2 studies), high-risk (2 studies), and the overall population (3 studies). The pooled analysis for the intermediate-to-high-risk subgroup showed no statistically significant OS benefit for PPaLND compared to PLND (HR = 0.62, 95% CI: 0.31, 1.24).Similarly, no significant benefit was found in the intermediate-risk or high-risk subgroups individually; however, the extremely wide confidence intervals (intermediate-risk: HR = 0.65, 95% CI: 0.01–44.47; high-risk: HR = 0.43, 95% CI: 0.05–4.04) reflect the very limited statistical power of these analyses, each based on only two studies, and should not be interpreted as evidence of no clinical benefit. Analysis of the overall population also failed to demonstrate a significant benefit. It is important to note, however, that in the analysis pooling data across all available subgroups, PPaLND showed a significant survival advantage (HR = 0.60, 95% CI: 0.44, 0.81). The forest plot for the adjusted analysis of PPaLND versus PLND is presented in **Figure 7**. Funnel plot analysis for this comparison indicated basic symmetry, suggesting no significant publication bias (**Figure 8**). As 9 studies contributed to this comparison, the funnel plot provides reasonably reliable evidence against significant publication bias, consistent with the observed basic symmetry.

##### 3.3.2.3 Sensitivity analysis

**Figure 9:**
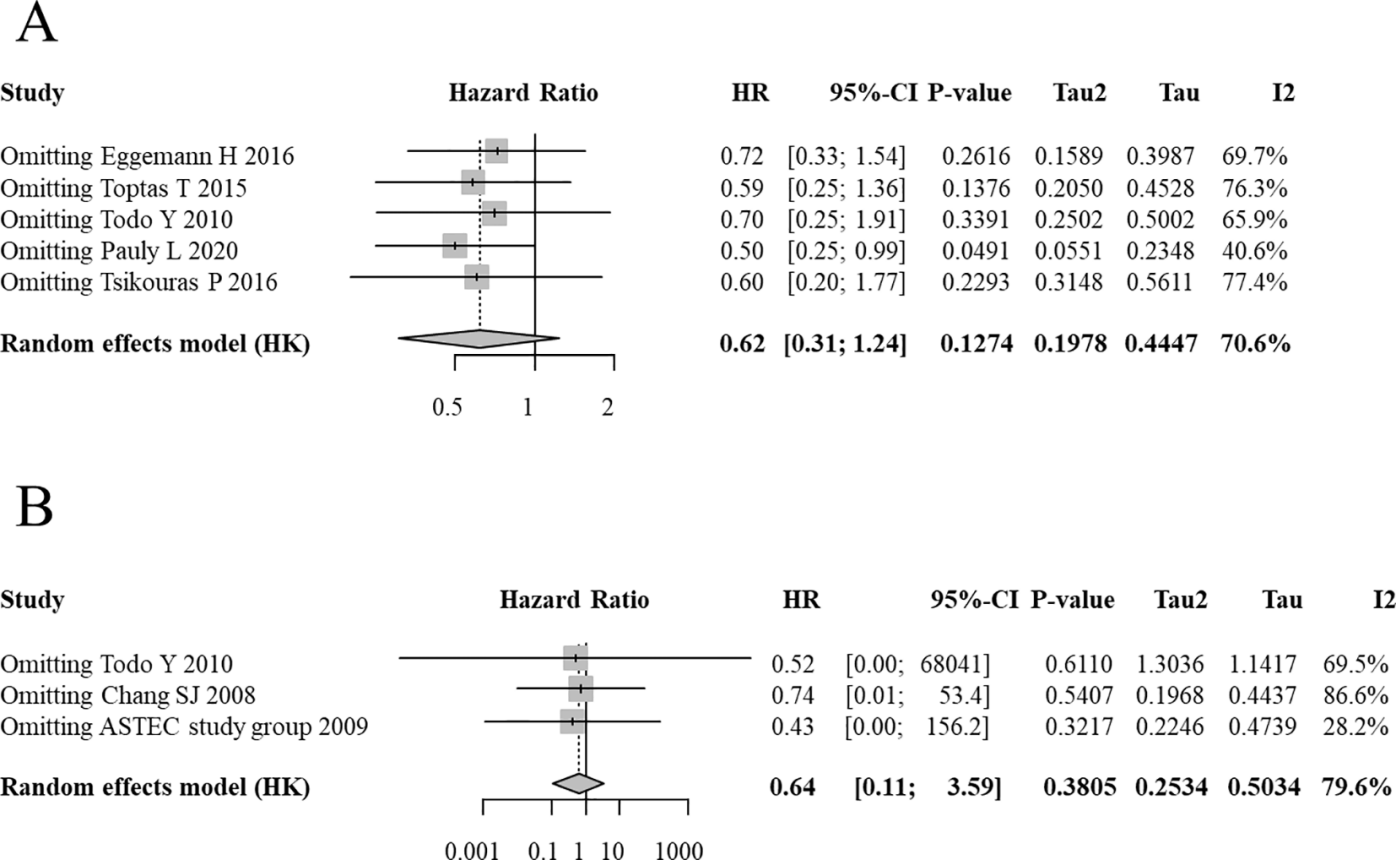
Sensitivity analysis forest plot for OS benefit of PPaLND versus pelvic (Adjusted; **A** Intermediate-to-high-risk subgroup; **B** Overall population subgroup.).

Leave-one-out sensitivity analysis for the intermediate-to-high-risk subgroup revealed that the exclusion of the study by Pauly L et al. (2020) (23) resulted in a statistically significant OS benefit for PPaLND (HR = 0.50, 95% CI: 0.25, 0.99), as shown in **Figure 9A**. Sensitivity analysis for the overall population subgroup continued to support the initial non-significant finding, as shown in **Figure 9B**.

## 4 Discussion

This systematic review and meta-analysis, incorporating 14 studies comprising 25, 432 patients with endometrial carcinoma, provides a comprehensive synthesis of evidence regarding the impact of lymphadenectomy extent on overall survival. The principal finding suggests that, after adjustment for potential confounders, combined PPaLND may be associated with a significant improvement in OS when compared to either no lymphadenectomy or PLND alone. However, this benefit was not consistently observed across risk-stratified subgroups: the intermediate-to-high-risk analysis showed no significant benefit (HR = 0.62, 95% CI: 0.31–1.24), and the intermediate- and high-risk subgroups each based on only two studies yielded extremely wide confidence intervals (HR = 0.65, 95% CI: 0.01–44.47; HR = 0.43, 95% CI: 0.05–4.04, respectively), precluding meaningful conclusions. Sensitivity analyses further demonstrated that several pooled estimates were sensitive to the exclusion of individual influential studies, indicating limited robustness. These findings suggest that the survival benefit of PPaLND may vary across risk populations and that the subgroup-specific conclusions should be considered hypothesis-generating rather than definitive. The substantial heterogeneity observed in the primary analyses (I² >70%) warrants careful consideration. We identified several likely contributors. First, patient selection criteria varied across studies, with different definitions of intermediate- and high-risk populations and inconsistent application of risk-stratification systems. Second, surgical extent was not standardized—para-aortic lymphadenectomy was defined variably, with some protocols specifying dissection to the renal vessels and others using more limited sampling. Third, adjuvant therapy protocols evolved over the 17-year study span (2008–2025), potentially modifying survival outcomes independently of surgical approach. Fourth, study design heterogeneity was prominent: 13 of 14 included studies were retrospective, and the covariates adjusted for in multivariable models varied considerably. These factors collectively limit the precision of the pooled estimates and suggest that the summary effect size represents an average across heterogeneous populations rather than a uniform treatment effect. Meta-regression to formally decompose these sources was precluded by the limited number of studies within each stratum.

A notable feature of our findings is the inconsistency between unadjusted and adjusted analyses. In both primary comparisons, unadjusted models did not demonstrate a significant OS benefit, whereas adjusted estimates showed a clear survival advantage. We designated adjusted estimates as the primary analysis because unadjusted comparisons in observational surgical studies are inherently confounded: clinicians selectively perform PPaLND in patients with more aggressive disease, biasing unadjusted results against the intervention group. Multivariable adjustment partially mitigates this confounding by indication but is itself subject to important limitations. The covariates adjusted for varied considerably across studies—only five of 14 simultaneously adjusted for both LVSI and adjuvant therapy—introducing model dependency. Moreover, unmeasured confounders such as surgeon experience, institutional volume, and patient performance status cannot be addressed by statistical adjustment. These residual sources of confounding are inherent to meta-analyses of retrospective observational data and underscore the need for prospective randomized trial validation. While our conclusions align broadly with prior meta-analyses, this study offers several incremental contributions: the largest pooled sample to date, explicit prioritization of adjusted estimates to address confounding by indication, systematic risk-stratified subgroup analyses, and transparent sensitivity analyses revealing result instability.

Our aggregate results align broadly with the conclusions of several prior meta-analyses and large-scale retrospective cohort investigations. For instance, a meta-analysis by Guo et al. (2018), which included 8 studies, demonstrated that for patients at intermediate-to-high risk of recurrence, PPaLND significantly improved OS compared to PLND alone (HR 0.52, 95% CI 0.39–0.69) (27). Similarly, Petousis et al. (2020), in an analysis of 13 studies, reported that PPaLND was associated with a 46% reduction in the risk of death and a 49% reduction in the risk of recurrence (28). The influential SEPAL study, a landmark retrospective cohort analysis, provided robust evidence that PPaLND significantly prolonged OS for intermediate- and high-risk patients (HR 0.53, 95% CI 0.38–0.76) (11). The failure of our analysis to replicate this statistically significant benefit within the intermediate-to-high-risk subgroup may be attributed to methodological variations, differences in the composition of the analyzed cohorts, and the pronounced heterogeneity observed among the included studies. It is noteworthy that a recent systematic review by Pavone et al. (2024), encompassing 14 studies and 9,415 patients, also found PPaLND to be associated with an improved 5-year overall survival rate (RR = 0.71, 95% CI 0.57–0.88), while simultaneously highlighting a 26% increased risk of postoperative complications (29). This underscores the critical concept that any potential survival gain from PPaLND must be carefully weighed against a concomitant increase in surgical morbidity, which may partially negate its net clinical benefit.

Significant controversy persists within the existing evidence base. The ASTEC trial (2009), a multicenter randomized controlled trial, concluded that systematic pelvic lymphadenectomy conferred no OS benefit for women with early-stage disease at intermediate or high risk (24). Furthermore, a study by Li et al. (2020) focusing specifically on type I endometrial carcinomas found no survival benefit associated with para-aortic lymphadenectomy (30). Several factors may account for these discrepant findings. First, there is a lack of uniform criteria for defining “intermediate-to-high risk” across studies, with inconsistencies in the assessment and reporting of key prognostic factors such as depth of myometrial invasion, histological grade, and lymphovascular space invasion (LVSI). Second, retrospective studies are inherently susceptible to indication bias, whereby surgeons may intuitively select patients with perceived worse prognoses (e.g., more aggressive tumor features intraoperatively) for more extensive lymphadenectomy. This bias is notoriously difficult to fully adjust for using statistical methods. Third, the evolution of adjuvant therapy paradigms over time—such as the shift from radiotherapy alone to chemoradiation or systemic chemotherapy—may influence survival outcomes independently, potentially masking or diluting the isolated therapeutic effect of lymph node dissection. Furthermore, only five of the 14 included studies adjusted for both lympho vascular space invasion and adjuvant therapy in their multivariable models (**Supplementary Table 1**), which may further contribute to the residual heterogeneity and limit the comparability of adjusted estimates across studies. Beyond these study-specific factors, the substantial heterogeneity observed in the primary analyses (I² >70%) reflects broader methodological and clinical diversity. Key contributors include variability in patient selection criteria and risk-stratification definitions, inconsistent surgical standards for para-aortic lymphadenectomy extent, evolving adjuvant therapy protocols over the 17-year study period (2008–2025), and the predominance of retrospective designs with differing adjustment strategies. Given this heterogeneity, the pooled estimates should be interpreted as an average effect across diverse populations rather than a precise, generalizable treatment effect. Meta-regression was not feasible due to the limited number of studies per stratum. Several characteristics of the Pauly et al. study may account for this influence. First, it is by far the largest study in this subgroup (n=2,030), thereby exerting considerable weight on the pooled estimate. Second, unlike the other studies which used overall survival as the endpoint. Pauly et al. reported disease-specific survival; the exclusion of non-cancer deaths may alter the estimated treatment effect, particularly in a population with a relatively advanced median age. Third, this study was based on the SEER population-level registry, which reflects real-world practice patterns and case mix that may differ from those of the primarily tertiary-center cohorts in the remaining studies. The apparent funnel plot asymmetry for the PPaLND versus control comparison should be interpreted with caution given the small number of studies, as noted in the results section.

The risk-benefit calculus for PPaLND must explicitly incorporate its associated morbidity profile. Lymphadenectomy, particularly when extending to the para-aortic region, is linked to a significantly elevated risk of both acute and chronic postoperative complications. Volpi et al. (2019) identified PPaLND as an independent predictor for lower limb lymphedema (OR = 2.764) and symptomatic lymphocyst formation (OR = 5.066) (31). Agar et al. (2015) reported a major complication rate of 17.5% and a perioperative mortality rate of 2.5% associated with lymphadenectomy. More recently, Terada et al. (2023) provided compelling comparative data, showing a markedly lower incidence of lower limb lymphedema following sentinel lymph node biopsy compared to systematic lymphadenectomy (2.0% vs. 21.3%) (32). A systematic review and meta-analysis by Helgers et al. (2020) further corroborates these findings, reporting that SLN biopsy was associated with significantly lower odds of any postoperative complication (pooled OR = 0.52, 95% CI: 0.36–0.73) and severe complications (pooled OR = 0.52, 95% CI: 0.28–0.96) compared with lymphadenectomy (12). These data compellingly argue that the therapeutic value of PPaLND cannot be assessed solely through the lens of survival metrics; a comprehensive evaluation must rigorously balance any potential survival advantage against the tangible risks of increased surgical morbidity and its impact on quality of life. Furthermore, the rapidly evolving landscape of molecular classification in endometrial cancer—including the identification of prognostically distinct subgroups defined by POLE ultramutated, mismatch repair-deficient (MMRd), p53 abnormal (p53abn), and no specific molecular profile (NSMP)—has fundamentally transformed risk stratification and treatment decision-making (33). Future prospective studies should integrate molecular classification into subgroup analyses to more precisely identify which patients are most likely to derive oncological benefit from extended lymphadenectomy, thereby promoting more individualized and molecularly informed surgical decision-making.

From a clinical practice perspective, the findings of this review hold significant implications for surgical strategy formulation in endometrial cancer. Current clinical practice guidelines, such as those from the European Society of Gynaecological Oncology (ESGO) and the National Comprehensive Cancer Network (NCCN), reflect the ongoing controversy, offering nuanced and sometimes divergent recommendations regarding the indications and extent of lymphadenectomy. Prospective, randomized trials currently underway, including the ECLAT trial (22) and the JCOG1412 trial (SEPAL-P3) (34), are poised to provide higher-level evidence to resolve this clinical equipoise. In the interim, and likely even thereafter, surgical decision-making should remain highly individualized. Clinicians must integrate a multitude of patient-specific factors—including detailed histopathological characteristics (histotype, grade, LVSI, depth of invasion), preoperative imaging findings, surgical fitness, and patient preferences—to perform a personalized assessment of the anticipated benefit-risk ratio of PPaLND for each individual.

### 4.1 Limitations

This study has several limitations that warrant consideration when interpreting its findings. First, the evidence base is predominantly retrospective; only one of the fourteen included studies was a randomized controlled trial (ASTEC, 2009), precluding formal subgroup analysis or meta-regression by study design. Retrospective studies are intrinsically vulnerable to selection bias and unmeasured confounding. Although the total sample size is large (25,432 patients), the number of included studies (n=14) is comparable to prior meta-analyses, indicating that the increase in sample size is driven by large retrospective datasets rather than improved evidence quality. This underscores that the expanded evidence base does not fundamentally resolve the methodological limitations inherent to retrospective data. Second, there was considerable variability in the technical definition of “para-aortic lymphadenectomy” across studies. The surgical upper limit varied, with some protocols mandating dissection up to the level of the renal vessels and others describing a more limited sampling. This lack of standardization in the intervention itself introduces clinical heterogeneity. Third, the definitions of ‘intermediate-risk’ and ‘high-risk’ varied considerably across studies, with some employing established classification systems (e.g., ESMO-ESGO-ESTRO) and others using author-defined criteria (**Supplementary Table X**). The small number of studies within each risk stratum precluded meaningful sensitivity analysis by classification system; this limitation should be considered when interpreting the risk-stratified results. Fourth, there was substantial variation in adjuvant treatment strategies across studies, including differences in the type (radiotherapy, chemotherapy, or combination), timing, and intensity of postoperative therapy. Given that survival outcomes are strongly influenced by adjuvant treatment decisions—which are themselves driven by surgical staging findings—it is difficult to isolate the independent effect of lymphadenectomy extent. This introduces important ambiguity in causal interpretation: the observed survival benefit may partly reflect differences in postoperative management rather than the surgical intervention itself. Furthermore, only five of the 14 included studies adjusted for adjuvant therapy in their multivariable models, limiting the ability to disentangle the treatment effect from confounding by adjuvant therapy. Sixth, the included studies did not incorporate molecular classification (POLE ultramutated, MMRd, p53abn, NSMP), which has become integral to contemporary risk stratification and treatment decision-making in endometrial cancer (33). The inability to assess whether the survival benefit of PPaLND varies by molecular subtype represents an important gap, and future prospective studies should integrate molecularly stratified subgroup analyses to permit more precise surgical decision-making.

## 5 Conclusion

In conclusion, this meta-analysis suggests that combined PPaLND may be associated with an overall survival benefit for patients with endometrial carcinoma. However, the magnitude and consistency of this benefit appear to vary across different risk-stratified populations. In particular, the intermediate-risk and high-risk subgroup analyses, each based on only two studies with extremely wide confidence intervals, should be considered exploratory and do not rule out a clinically meaningful benefit. The decision to perform an extended lymphadenectomy necessitates a careful, individualized assessment that weighs a potential, and often uncertain, survival advantage against a well-documented increase in surgical morbidity. Future research efforts should be directed towards the precise identification of patient subgroups that derive the greatest oncological benefit from PPaLND, thereby enabling a more targeted application of this procedure. Concurrently, rigorous evaluation of less morbid staging strategies, such as sophisticated sentinel lymph node biopsy algorithms and molecular profiling for risk stratification, is imperative. The goal is to develop management paradigms that optimize survival outcomes while simultaneously minimizing treatment-related toxicity and preserving quality of life.
