# Supplementary material for: Survival benefit of extended lymphadenectomy in endometrial cancer: a meta-analysis with risk-stratified subgroup analysis
Source: Front Oncol. 2026 Jun 10;16:1835770. doi: 10.3389/fonc.2026.1835770 (PMC13290997; doi:10.3389/fonc.2026.1835770)
Supplement: Supplementary file 1 [file Table1.docx]

Supplementary Table 1: Adjustment Variables Used in Multivariable Analyses of Included Studies

| **Study (Year)** | **Age** | **Stage** | **Grade** | **Histology** | **LVSI** | **MI depth** | **Tumor size** | **Adjuvant therapy** | **Other covariates** |
| --- | --- | --- | --- | --- | --- | --- | --- | --- | --- |
| Eggemann 2016 | ✓ | ✓ | ✓ | ✓ | — | ✓ | — | — | — |
| Toptas 2015 | ✓ | ✓ | ✓ | — | ✓ | ✓ | — | ✓ | — |
| Tong 2011 | ✓ | — | — | — | — | — | — | — | Parity, BMI, tumor diameter, No. of pelvic LN |
| Todo 2010 | ✓ | ✓ | ✓ | ✓ | — | ✓ | — | ✓ | Cervical invasion |
| Chang 2008 | ✓ | ✓ | ✓ | ✓ | ✓ | ✓ | — | ✓ | Cervical invasion, cytology |
| Vatansever 2020 | ✓ | ✓ | — | — | — | — | — | ✓ | LN metastasis |
| Yalcin 2025 | ✓ | ✓ | — | ✓ | ✓ | ✓ | — | — | Cytology, surgical approach |
| **Seagle 2017** | ✓ | ✓ | ✓ | — | ✓ | ✓ | ✓ | **✓ (RT+CT)** | Race, insurance, comorbidity, margin status, hospital volume, prior cancer |
| Lai 2025 | ✓ | ✓ | ✓ | — | ✓ | ✓ | — | — | Cervical invasion |
| Papathemelis 2017 | ✓ | ✓ | — | ✓ | — | — | — | — | Year of diagnosis, lymph/vein invasion, region of LND |
| **Pauly 2020** | **✓ (matched)** | — | — | — | — | — | — | — | Matched by age and risk of recurrence only |
| **ASTEC 2009** | ✓ | — | — | — | — | — | — | — | WHO PS, weeks to surgery, surgical technique, incision type |
| **Venigalla 2018** | ✓ | ✓ | ✓ | ✓ | ✓ | ✓ | ✓ | **✓ (RT+CT)** | Race, insurance, education, facility type, comorbidity, margin status (PS-weighted) |
| Tsikouras 2016 | ✓ | — | — | — | — | — | — | — | Multivariate for OS and DSS; specific covariates not fully listed |

**Abbreviations:** MI = myometrial invasion; LVSI = lymphovascular space invasion; LN = lymph node; PS = propensity score; RT = radiotherapy; CT = chemotherapy; WHO PS = World Health Organization performance status; ✓ = variable included in multivariable model; — = not included or not reported.
